# Flapless versus Conventional Flapped Dental Implant Surgery: A Meta-Analysis

**DOI:** 10.1371/journal.pone.0100624

**Published:** 2014-06-20

**Authors:** Bruno Ramos Chrcanovic, Tomas Albrektsson, Ann Wennerberg

**Affiliations:** 1 Department of Prosthodontics, Faculty of Odontology, Malmö University, Malmö, Sweden,; 2 Department of Biomaterials, Göteborg University, Göteborg, Sweden; University of Toronto, Canada

## Abstract

The aim of this study was to test the null hypothesis of no difference in the implant failure rates, postoperative infection, and marginal bone loss for patients being rehabilitated by dental implants being inserted by a flapless surgical procedure versus the open flap technique, against the alternative hypothesis of a difference. An electronic search without time or language restrictions was undertaken in March 2014. Eligibility criteria included clinical human studies, either randomized or not. The search strategy resulted in 23 publications. The I^2^ statistic was used to express the percentage of the total variation across studies due to heterogeneity. The inverse variance method was used for random-effects model or fixed-effects model, when indicated. The estimates of relative effect were expressed in risk ratio (RR) and mean difference (MD) in millimeters. Sixteen studies were judged to be at high risk of bias, whereas two studies were considered of moderate risk of bias, and five studies of low risk of bias. The funnel plots indicated absence of publication bias for the three outcomes analyzed. The test for overall effect showed that the difference between the procedures (flapless vs. open flap surgery) significantly affect the implant failure rates (*P* = 0.03), with a RR of 1.75 (95% CI 1.07–2.86). However, a sensitivity analysis revealed differences when studies of high and low risk of bias were pooled separately. Thus, the results must be interpreted carefully. No apparent significant effects of flapless technique on the occurrence of postoperative infection (P = 0.96; RR 0.96, 95% CI 0.23–4.03) or on the marginal bone loss (*P* = 0.16; MD −0.07 mm, 95% CI −0.16–0.03) were observed.

## Introduction

When placing dental implants, a flap is traditionally elevated to better visualize the implant recipient site, providing that some anatomical landmarks are clearly identified and protected. When a limited amount of bone is available, a flap elevation can help implant placement to reduce the risk of bone fenestrations or perforations [1]. More recently, the concept of flapless implant surgery has been introduced for the patients with sufficient keratinized gingival tissue and bone volume in the implant recipient site. In a flapless procedure, a dental implant is installed through the mucosal tissues without reflecting a flap. The alleged reasons to choose the flapless technique are to minimize the possibility of postoperative peri-implant tissue loss and to overcome the challenge of soft tissue management during or after surgery [2]. Other alleged advantages of the flapless implant surgery include less traumatic surgery, decreased operative time, rapid postsurgical healing, fewer postoperative complications and increased patient comfort [3], [4]. A disadvantage of this technique is that the true topography of the underlying available bone cannot be observed because the mucogingival tissues are not raised, which may increase the risk for unwanted perforations which in its turn could lead to esthetical problems or implant losses [5]. Moreover, there is the potential for thermal damage secondary to reduced access for external irrigation during osteotomy preparation [4].

Researchers have been trying to evaluate whether the insertion of implants by the flapless technique may influence the survival of dental implants. However, some studies may lack statistical power, given the small number of patients per group in the clinical trials comparing the techniques. Thus, we conducted a meta-analysis of previously published clinical studies to investigate whether there are any positive effects of flapless implant insertion surgery on implant failure rates, postoperative infection, and marginal bone loss in comparison with the more traditional open flap technique. The present study presents a more detailed and profound analysis of the influence of these two techniques on the implant failure rates, previously assessed in a published systematic review [6].

## Materials and Methods

This study followed the PRISMA Statement guidelines [7]. A review protocol does not exist.

### Objective

The purpose of the present review was to test the null hypothesis of no difference in the implant failure rates, postoperative infection, and marginal bone loss for patients being rehabilitated by dental implants being inserted by a flapless surgical procedure versus the open flap technique, against the alternative hypothesis of a difference.

### Search strategies

An electronic search without time or language restrictions was undertaken in March 2014 in the following databases: PubMed, Web of Science, and the Cochrane Oral Health Group Trials Register. The following terms were used in the search strategy on PubMed:

{Subject AND Adjective}

{*Subject*: (dental implant OR dental implant failure OR dental implant survival OR dental implant success [text words])

AND

*Adjective*: (flapless OR flapped OR open flap [text words])}

Refining the results with the option “Dentistry Oral Surgery Medicine” selected within the filter “Research Areas”, the following terms were used in the search strategy on Web of Science:

{Subject AND Adjective}

{*Subject*: (dental implant failure OR dental implant survival OR dental implant success [title])

AND

*Adjective*: (flapless surgery OR flapped surgery OR open flap surgery [title])}

The following terms were used in the search strategy on the Cochrane Oral Health Group Trials Register:

(dental implant OR dental implant failure OR dental implant survival OR dental implant success AND (flapless surgery OR flapped surgery OR open flap surgery))

A manual search of dental implants-related journals, including *British Journal of Oral and Maxillofacial Surgery, Clinical Implant Dentistry and Related Research, Clinical Oral Implants Research, European Journal of Oral Implantology, Implant Dentistry, International Journal of Oral and Maxillofacial Implants, International Journal of Oral and Maxillofacial Surgery, International Journal of Periodontics and Restorative Dentistry, International Journal of Prosthodontics, Journal of Clinical Periodontology, Journal of Dental Research, Journal of Oral Implantology, Journal of Craniofacial Surgery, Journal of Cranio-Maxillofacial Surgery, and Journal of Maxillofacial and Oral Surgery, Journal of Oral and Maxillofacial Surgery, Journal of Periodontology,* and *Oral Surgery Oral Medicine Oral Pathology Oral Radiology and Endodontology*, was also performed.

The reference list of the identified studies and the relevant reviews on the subject were also scanned for possible additional studies. Moreover, online databases providing information about clinical trials in progress were checked (clinicaltrials.gov; www.centerwatch.com/clinicaltrials; www.clinicalconnection.com).

### Inclusion and Exclusion Criteria

Eligibility criteria included clinical human studies, either randomized or not, comparing implant failure rates in any group of patients receiving titanium dental implants by a flapless surgical procedure versus the open flap technique. For this review, implant failure represents the complete loss of the implant. Exclusion criteria were case reports, technical reports, animal studies, *In Vitro* studies, and reviews papers.

### Study selection

The titles and abstracts of all reports identified through the electronic searches were read independently by the three authors. For studies appearing to meet the inclusion criteria, or for which there were insufficient data in the title and abstract to make a clear decision, the full report was obtained. Disagreements were resolved by discussion between the authors.

### Quality assessment

The quality assessment was performed by using the recommended approach for assessing risk of bias in studies included in Cochrane reviews [8]. The classification of the risk of bias potential for each study was based on the four following criteria: sequence generation (random selection in the population), allocation concealment (steps must be taken to secure strict implementation of the schedule of random assignments by preventing foreknowledge of the forthcoming allocations), incomplete outcome data (clear explanation of withdrawals and exclusions), and blinding (measures to blind study participants and personnel from knowledge of which intervention a participant received). The incomplete outcome data will also be considered addressed when there are no withdrawals and/or exclusions. A study that met all the criteria mentioned above was classified as having a low risk of bias, a study that did not meet one of these criteria was classified as having a moderate risk of bias. When two or more criteria were not met, the study was considered to have a high risk of bias.

### Data extraction and meta-analysis

From the studies included in the final analysis, the following data was extracted (when available): year of publication, study design (randomized controlled trial – RCT, controlled clinical trial – CCT, retrospective study), unicenter or multicenter study, number of patients, patients' age, follow-up, days of antibiotic prophylaxis, mouth rinse with chlorhexidine, implant healing period, failed and placed implants, postoperative infection, marginal bone loss, implant surface modification, use of grafting procedures, use of a surgical guide, and presence of smokers among the patients. Contact with authors for possible missing data was performed.

Implant failure and postoperative infection were the dichotomous outcomes measures evaluated. Weighted mean differences were used to construct forest plots of marginal bone loss, a continuous outcome. The statistical unit for ‘implant failure’ and ‘marginal bone loss’ was the implant, and for ‘postoperative infection’ was the patient. Whenever outcomes of interest were not clearly stated, the data were not used for analysis. The I^2^ statistic was used to express the percentage of the total variation across studies due to heterogeneity, with 25% corresponding to low heterogeneity, 50% to moderate and 75% to high. The inverse variance method was used for random-effects or fixed-effects model. Where statistically significant (*P*<.10) heterogeneity is detected, a random-effects model was used to assess the significance of treatment effects. Where no statistically significant heterogeneity is found, analysis was performed using a fixed-effects model [9]. In the inverse variance method the weight given to each study is chosen to be the inverse of the variance of the effect estimate (i.e. one over the square of its standard error) [8]. Thus larger studies, which have smaller standard errors, are given more weight than smaller studies, which have larger standard errors. This choice of weight minimizes the imprecision (uncertainty) of the pooled effect estimate. The basic data required for the analysis are an estimate of the intervention effect and its standard error from each study [8].

The estimates of relative effect for dichotomous outcomes were expressed in risk ratio (RR) and in mean difference (MD) in millimeters for continuous outcomes, both with a 95% confidence interval (CI). Only if there were studies with similar comparisons reporting the same outcome measures was meta-analysis to be attempted. In the case where no events (or all events) are observed in both groups the study provides no information about relative probability of the event and is automatically omitted from the meta-analysis. In this (these) case(s), the term ‘not estimable’ is shown under the RR column of the forest plot table. The software used here automatically checks for problematic zero counts, and adds a fixed value of 0.5 to all cells of study results tables where the problems occur.

A funnel plot (plot of effect size versus standard error) will be drawn. Asymmetry of the funnel plot may indicate publication bias and other biases related to sample size, although the asymmetry may also represent a true relationship between trial size and effect size.

The data were analyzed using the statistical software Review Manager (version 5.2.8, The Nordic Cochrane Centre, The Cochrane Collaboration, Copenhagen, Denmark, 2014).

## Results

### Literature search

**Figure 1:**
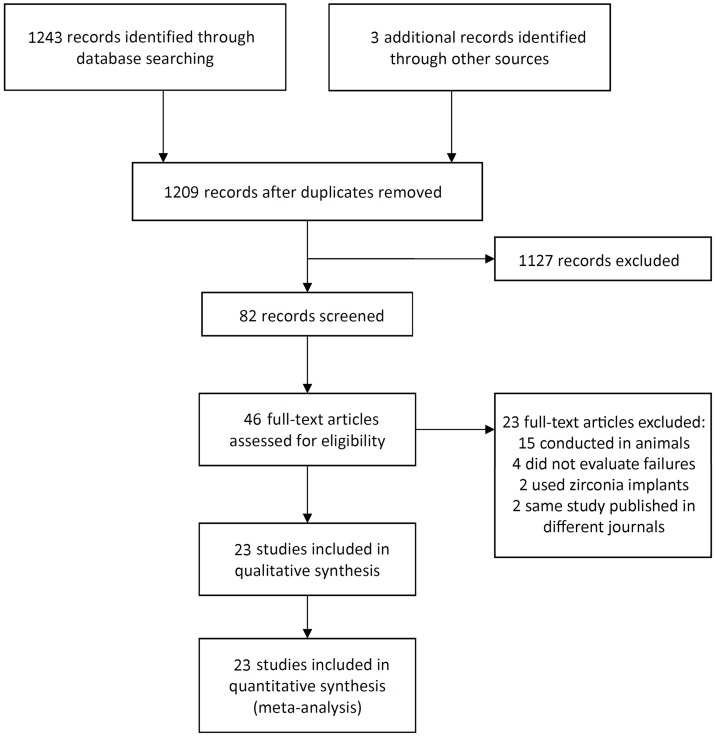
Study screening process – flow diagram.

The study selection process is summarized in Figure 1. The search strategy resulted in 1246 papers. The three reviewers independently screened the abstracts for those articles related to the focus question. The initial screening of titles and abstracts resulted in 82 full-text papers; 37 were cited in more than one research of terms. The full-text reports of the remaining 43 articles led to the exclusion of 23 because they did not meet the inclusion criteria; 15 studies were conducted in animals, 2 studies used zirconia implants, 4 studies compared the techniques but did not evaluate implant failures, and 2 articles were the same study published in different journals. Additional hand-searching of the reference lists of selected studies yielded two additional papers. Thus, a total of 23 publications were included in the review.

### Description of the Studies

Detailed data of the 23 included studies are listed in Table 1. Ten RCTs [1], [4], [10]–[17], seven CCTs [3], [18]–[23] and six retrospective studies [5], [24]–[28] were included in the meta-analysis. Only three studies [1], [25], [27] were multicenter. In five studies [4], [12]–[14], [17] both patients and operators/outcome assessors were blinded to the tested intervention. Six studies [3], [11], [12], [16], [17], [21] had a follow-up up to 6 months, and six studies [15], [18]–[20], [22], [23] with a follow-up up to 1 year.

**Table 1:** Detailed data of the included studies.

Authors	Published	Patients (n) (number per group)	Patients' Age Range (Average) (years)	Follow-up visits (or range)	Failed/Placed Implants (n)	Implant failure rate (%)	*P* value (for failure rate)	Antibiotics/mouth rinse (days)	Healing period/loading	Implant surface modification (brand)	Grafting	Observations
Kinsel and Liss [24]	2007	43 (NM)	35–80 (58)	2–10 years	13/196 (G1) 3/148 (G2)	6.6 (G1) 2.0 (G2)	0.07	NM	Immediate	TPS (SLA, Straumann, Basel, Switzerland; n = 131), sandblasted and acid-etched (SLA, Straumann, Basel, Switzerland; n = 213)	Grafting in 2 patients, with implants placed 5–6 months later	12 smokers, surgical guide (G1 and G2)
Nkenke et al. [18]	2007	10 (5, G1, 5, G2)	NM (65±10)	1 and 7 days, 12 months	0/30 (G1) 0/30 (G2)	0 (G1) 0 (G2)	NM	NM	6 months	NM	NP	Only in maxilla, use of CT-guided surgical stents (G1)
Ozan et al. [1]	2007	12 (5, G1; 7, G2)	NM (46±9)	6–14 months (mean 9±3)	0/14 (G1) 1/45 (G2)	0 (G1) 2.2 (G2)	NM	NM	3 months (maxilla) 2 months (mandible)	Sandblasted and acid-etched (SwissPlus, Zimmer Dental, Carlsbad, USA)	NP	Use of CT-guided surgical stents (G1), healing abutments screwed immediately
Villa and Rangert [19]	2007	33 (15, G1; 18, G2)	NM	10 days, 1, 3, 6 and 12 months	1/29 (G1) 1/47 (G2)	3.4 (G1) 2.1 (G2)	NM	6/14–21	Immediate and early loading	Oxidized (Brånemark Mk III and Mk IV and NobelSpeedy, TiUnite, Nobel Biocare, Göteborg, Sweden)	NP	Implants placed in infected extraction sockets, no use of a surgical guide
Cannizzaro et al. [10]	2008	40 (20, G1; 20, G2)	18–62 (40.1, G1) 19–64 (37.4, G2)	3 years	0/52 (G1) 0/56 (G2)	0 (G1) 0 (G2)	NM	3/13	Immediate loading (G1), 4 months (maxilla) and 3 months (mandible) (G2)	Sandblasted and acid-etched (SwissPlus, Zimmer Dental, Carlsbad, USA)	NP	Use of surgical templates based on diagnostic tooth arrangement (G1), 17 smokers (8, G1; 9, G2)
Covani et al. [11]	2008	20 (10, G1; 10, G2)	30–67 (NM)	6 months	1/10 (G1) 0/10 (G2)	10 (G1) 0 (G2)	NM	4/21	6 months	Titanium plasma-sprayed coated (Premium, Sweden & Martina, Padova, Italy)	All implants: grafting with a mixture of collagen gel/corticocancellous porcine bone	Submerged implants, no use of a surgical guide, heavy smokers (>10 cigarettes/day) were excluded
Maló and Nobre [20]	2008	41 (20, G1; 21, G2)	19–79 (45.5)	6 months 1 year	1/32 (G1) 0/40 (G2)	3.1 (G1) 0 (G2)	NM	6/60 (rinse with hyaluronic acid)	Immediate loading	Oxidized (NobelSpeedy, TiUnite, Nobel Biocare, Göteborg, Sweden)	NP	No use of a surgical guide
Sennerby et al. [25]	2008	43 (NM)	NM (50)	1–18 months (mean 10.2)	6/76 (G1) 0/41 (G2)	7.9 (G1) 0 (G2)	NM	NM	Immediate/early (n = 95), from 6 weeks to 6 months (n = 22)	Anodically oxidized (NobelDirect, Nobel Biocare, Göteborg, Sweden)	Minor bone grafting in 8 implants	Use of a slide-over guide sleeve to evaluate and determine the position of the implant (G1) Healed sites (n = 99) Extraction sockets (n = 18)
Danza et al. [26]	2009	93 (8, G1; 85, G2)	16–89 (48)	Mean of 14 months	0/66 (G1) 9/225 (G2)	0 (G1) 4.0 (G2)	0.3311	5/NP	Immediate or after 3 months	? (3D Alpha-Biomedical s.r.l., Pescara, Italy)	NP	Use of CT-guided surgical template (G1), heavy smokers (>20 cigarettes/day) were excluded
Arisan et al. [3]	2010	52 (15, G1; 37, G2)	28–63 (48.4)	4 months	3/99 (G1) 5/242 (G2)	3.0 (G1) 2.1 (G2)	0.946	5/before surgery	2–4 months	Sandblasted and acid-etched (SPI-Element, Thommen Medical, Waldenburg, Switzerland, n = 180), sandblasted and acid-etched (XiVe, Dentsply-Friadent, Mannheim, Germany, n = 161)	NP	Use of a stereolithographic surgical guide (G1) In G2: the surgical guide was used in 16 patients (101 implants), whereas in 21 patients it was not used (141 implants), heavy smokers (>10 cigarettes/day) were excluded
Berdougo et al. [27]	2010	169 (99, G1; 76 G2)^a^	20–84 (53.1±14.5)	1–4 years	10/271 (G1) 4/281 (G2)	3.7 (G1) 1.4 (G2)	0.1	NM	NM	NM	NP	Use of an image-guided template (G1), 22 patients were smokers
Lindeboom and van Wijk [12]	2010	16 (8, G1; 8, G2)	NM (54.6±2.9, G1) NM (58.7±7.2, G2)	1 week 1 month 6 months b	3/48 (G1) 0/48 (G2)^b^	4.2 (G1) 0 (G2)	NM	5/7	The implants were not loaded	Oxidized (NobelReplace, Nobel Biocare, Göteborg, Sweden)	NP	Use of CT-guided surgical template (G1 and G2) No smokers
Rousseau [28]	2010	219 (121, G1; 98, G2)	23–84 (54.3±12.6)	4 weeks, 2–3 months, 2 years	3/174 (G1) 3/203 (G2)	1.7 (G1) 1.5 (G2)	0.46	NM	2–3 months	Sandblasted and acid-etched (SLA, Straumann, Basel, Switzerland)	NP	No use of a surgical guide
Van de Velde et al. [13]	2010	14 (split-mouth design)	39–75 (55.7)	1 week, 6 weeks, 3, 6, 12 and 18 months	1/36 (G1) 0/34 (G2)	2.8 (G1) 0 (G2)	NM	NP/before surgery	Immediate loading (G1), 6 weeks (G2)	Sandblasted and acid-etched (SLA, Straumann, Basel, Switzerland)	Bone grafts/sinus lifts: performed with a minimum of 6 months before implant installation	Use of a stereolithographic surgical guide (G1), heavy smokers (>10 cigarettes/day) were excluded
Cannizzaro et al. [14]	2011	40 (split-mouth design)	22–65 (44.5)	3 days, 10 days, 6 weeks, 8 weeks Every 3 months for 1 year	2/76 (G1) 2/67 (G2)	2.6 (G1) 3.0 (G2)	1.0	Preoperative/12	Immediate loading	Sandblasted and acid-etched (SwissPlus, Zimmer Dental, Carlsbad, USA)	NP	Nonsubmerged implants, 49 extraction sockets implants (25, G1; 24, G2), no use of a surgical guide, 20 patients were smokers
De Bruyn et al. [5]	2011	49 (NM)	20–79 (53)	1 and 3 years	0/28 (G1) 0/25 (G2)	0 (G1) 0 (G2)	NM	7/unknown number of days	3–6 months	Porous anodized surface (TiUnite, Nobel Biocare, Göteborg, Sweden)	NP	No use of a surgical guide, 10 patients were smokers
Froum et al. [15]	2011	52 (60)^c^ (27, G1; 25, G2)	NM	6 months, 1 year	0/27 (G1) 0/25 (G2)	0 (G1) 0 (G2)	NM	7/NM	8–12 weeks	Anodically oxidized (NobelDirect, Nobel Biocare, Göteborg, Sweden)	NP	Use of a surgical guide (G1 and G2)
Al-Juboori et al. [16]	2012	9 (split-mouth design)	27–62 (50)	6 and 12 weeks	0/11 (G1) 0/11 (G2)	0 (G1) 0 (G2)	NM	Before surgery/before surgery	The implants were not loaded	Sandblasted and acid-etched (SLA, Straumann, Basel, Switzerland)	NP	No use of a surgical guide
Katsoulis et al. [21]	2012	40 (17, G1; 23, G2)	47–78 (61±9)	1 week 3 months	0/85 (G1) 0/110 (G2)	0 (G1) 0 (G2)	NM	5/unknown number of days	The implants were not loaded	Oxidized (NobelReplace Select Tapered, Nobel Biocare, Göteborg, Sweden)	NP	Use of a stereolithographic surgical guide (G1), 3 patients were light smokers
Marcelis et al. [22]	2012	29 (NM)	NM (48.7±16.4)	1 year of functional loading	0/16 (G1) 1/18 (G2) d	0 (G1) 5.6 (G2)	NM	NM	Immediate (n = 9) 2–3 months (n = 24) ≥ 6 months (n = 1)	Sandblasted + fluoride (Osseospeed, AstraTech, Mölndal, Sweden)	NP	Use of a surgical guide (G1 and G2), 3 patients were smokers
Sunitha and Sapthagiri [4]	2013	40 (20, G1; 20, G2)	25–62 (39±4)	1 week, 3 and 6 months, 1 and 2 years	0/20 (G1) 0/20 (G2)	0 (G1) 0 (G2)	NM	5/NP	NM	NM	NP	No use of a surgical guide No smokers
Tsoukaki et al. [17]	2013	20 (10, G1; 10, G2)	30–62 (47)	1, 2, 6, and 12 weeks	0/15 (G1) 0/15 (G2)	0 (G1) 0 (G2))	NM	4/15	The implants were not loaded	Sandblasted + fluoride (Osseospeed, Astra Tech Dental, Mölndal, Sweden)	NP	Nonsubmerged implants, use of surgical guides (G1 and G2), heavy smokers (>10 cigarettes/day) were excluded
Meizi et al. [23]	2014	155 (NM)	NM (47.5)	3–9 months	7/237 (G1) 3/107 (G2)	2.95 (G1) 2.80 (G2)	NM	5/NM	Immediate (155, G1; 29, G2) 3–6 months (160)	Sandblasted and acid-etched (Saturn, Cortex Dental, Shlomi, Israel)	NM	No use of a surgical guide, 7% of the patients were diabetics, and 8% were smokers, 215 implants in fresh extraction sockets

NM – not mentioned; G1 – group flapless surgery; G2 – group conventional flapped surgery; NP – not performed

a The total of patients does not equal 169 because of cases treated with both protocols or in two phases of treatment in different years.

b Unpublished information concerning the number of failed implants in each group was obtained by personal communication with one of the authors. In this case 3 implants were lost in the flapless group at the 6-month follow-up

c There were 60 patients at the beginning of the study, but only 52 completed the study with 1 year of follow-up

d Unpublished information concerning the number of failed implants in each group was obtained by personal communication with one of the authors.

All studies but one [26] with available data of patients' age included only adult patients. Three split-mouth design studies were performed [13], [14], [16]. Eight studies [1], [10], [13], [18], [21], [25]–[27] made use of surgical guides when inserting implants through the flapless surgical technique, five studies [12], [15], [17], [22], [24] used the surgical guides in both groups, whereas in ten studies [3]–[5], [11], [14], [16], [19], [20], [23], [28] the implants were inserted without any kind of surgical guide.

Not every article provided information about the number of failed implants or to which group the failed implants belonged to. Unpublished information concerning the number of failed implants in each group was obtained by personal communication with one of the authors in two studies [12], [22]. From the 23 studies, a total of 1648 implants were placed through the flapless technique, with 51 failures (3.09%), and 1848 implants were placed through an open flap surgery, with 32 failures (1.73%). Nine studies [1], [11]–[13], [19], [20], [22], [23], [25] did not inform whether there was a statistically significant difference or not between the techniques concerning implant failure, whereas the other six studies [3], [14], [24], [26]–[28] did not find statistically significant difference. There were no implant failures in eight studies [4], [5], [10], [15]–[18], [21].

Thirteen articles [1], [3]–[5], [11], [13], [15], [22]–[27] did not report the incidence of postoperative infection. From the ten studies [10], [12], [14], [16]–[21], [28] that provided this information, it was observed 3 occurrences of infection in 265 patients receiving implants through the flapless technique (1.1%), and 3 episodes of postoperative infection in 252 patients receiving implants through the open flap surgery (1.2%).

### Quality Assessment

Each trial was assessed for risk of bias, and the scores are summarized in Table 2. Sixteen studies were judged to be at high risk of bias [1], [3], [5], [11], [16], [18]–[28], whereas two studies were considered of moderate risk of bias [10], [15], and five studies of low risk of bias [4], [12]–[14], [17].

**Table 2:** Results of quality assessment.

Authors	Published	Sequence generation (randomized?)	Allocation concealment	Incomplete outcome data addressed	Blinding	Estimated potential risk of bias
Kinsel and Liss [24]	2007	No	Inadequate	No	No	High
Nkenke et al. [18]	2007	No	Inadequate	No	No	High
Ozan et al. [1]	2007	Yes	Unclear	Yes	Unclear	High
Villa and Rangert [19]	2007	No	Inadequate	Yes	No	High
Cannizzaro et al. [10]	2008	Yes	Adequate	Yes	No	Moderate
Covani et al. [11]	2008	Yes	Unclear	Yes	No	High
Maló and Nobre [20]	2008	No	Inadequate	Yes	No	High
Sennerby et al. [25]	2008	No	Inadequate	Yes	No	High
Danza et al. [26]	2009	No	Inadequate	No	No	High
Arisan et al. [3]	2010	No	Inadequate	Yes	No	High
Berdougo et al. [27]	2010	No	Inadequate	No	No	High
Lindeboom and van Wijk [12]	2010	Yes	Adequate*	Yes*	Yes*	Low
Rousseau [28]	2010	No	Inadequate	No	No	High
Van de Velde et al. [13]	2010	Yes	Adequate	Yes	Yes	Low
Cannizzaro et al. [14]	2011	Yes	Adequate	Yes	Yes	Low
De Bruyn et al. [5]	2011	No	Inadequate	Yes	No	High
Froum et al. [15]	2011	Yes	Adequate	Yes	Unclear	Moderate
Al-Juboori et al. [16]	2012	Yes	Inadequate	Yes*	No	High
Katsoulis et al. [21]	2012	No	Inadequate	No	No	High
Marcelis et al. [22]	2012	No	Inadequate	Yes	No	High
Sunitha and Sapthagiri [4]	2013	Yes	Adequate	Yes	Yes	Low
Tsoukaki et al. [17]	2013	Yes	Adequate	Yes	Yes	Low
Meizi et al. [23]	2014	No	Inadequate	No	No	High

* Unpublished information was obtained by personal communication with one of the authors.

### Meta-analysis

In this study, a fixed-effects model was used to evaluate the implant failure, since statistically significant heterogeneity was not found (*P* = 0.86; I^2^  = 0%). The fixed-effects model was also used when the postoperative infection outcomes were evaluated, because statistically significant heterogeneity was also not found (*P* = 0.58; I^2^  = 0%).

**Figure 2:**
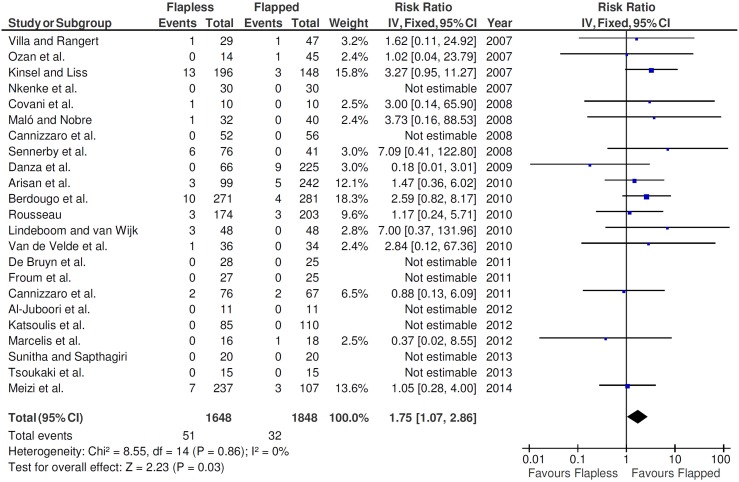
Forest plot of comparison of flapless versus open flap surgery for the event ‘implant failure’.

The test for overall effect showed that the difference between the procedures (flapless vs. flapped) statistically affected the implant failure rates (*P* = 0.03; Figure 2). A RR of 1.75 (95% CI 1.07–2.86) for the use of flapless surgery implies that failures when implants are inserted by the flapless surgery are 1.75 times likely to happen than failures when implants are inserted by the open flap technique. Thus, the relative risk reduction (RRR) is −75%. In other words, being the RRR negative, the insertion of implants by the flapless surgery increases the risk of implant failure by 75%. Since the RR could differ depending on the risk of bias of the studies, a sensitivity analysis was performed. The RR was examined for the groups of studies of low and high risk of bias. The reasons to not include studies of moderate risk of bias was that there were only two studies [10], [15], and no events were observed in both. When all low risk of bias studies were pooled, a RR of 1.84 resulted (95% CI 0.44–7.77; *P* = 0.49; I^2^  = 0%), whereas when all high risk of bias studies were pooled, a RR of 1.73 was observed (95% CI 1.03–2.93; *P* = 0.04; I^2^  = 0%).

**Figure 3:**
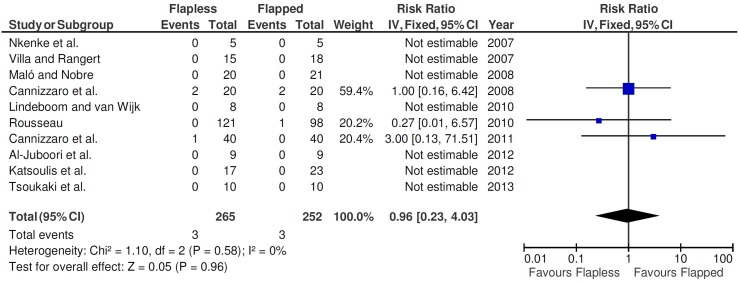
Forest plot of comparison of flapless versus open flap surgery for the event ‘postoperative infection’.

On the other side, the meta-analysis showed that there are no apparent significant effects of flapless surgery on the occurrence of postoperative infection in patients receiving implants (RR 0.96, 95% CI 0.23–4.03; *P* = 0.960; Figure 3).

**Figure 4:**
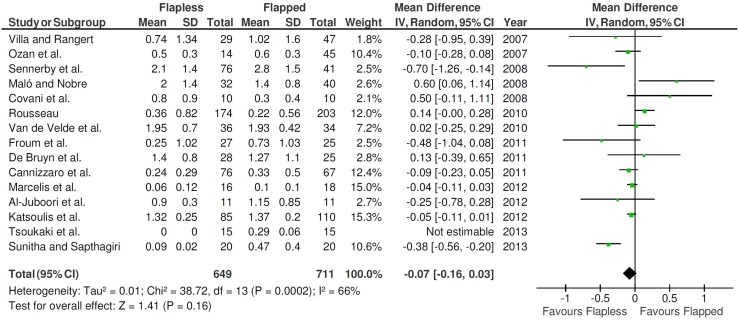
Forest plot of comparison of immediate nonfunctional versus immediate functional loading for the event ‘marginal bone loss’ (values in millimeters).

Fifteen studies (1360 implants) provided information about the marginal bone loss with standard deviation, necessary for the calculation of comparisons in continuous outcomes (Figure 4). A random-effects model was used to evaluate the marginal bone loss, since statistically significant heterogeneity was found (*P* = 0.0002; I^2^  = 66%). There was no statistically significant difference (*P* = 0.16) between the different techniques concerning the marginal bone loss.

### Publication bias

**Figure 5:**
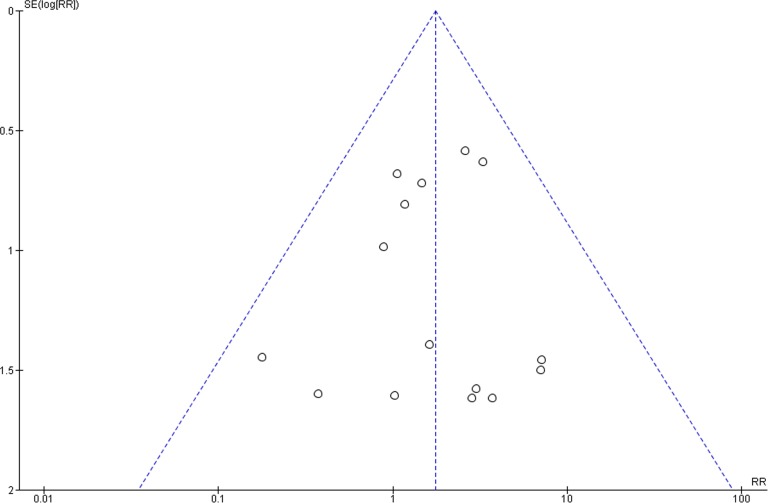
Funnel plot for the studies reporting the outcome event ‘implant failure’.

**Figure 6:**
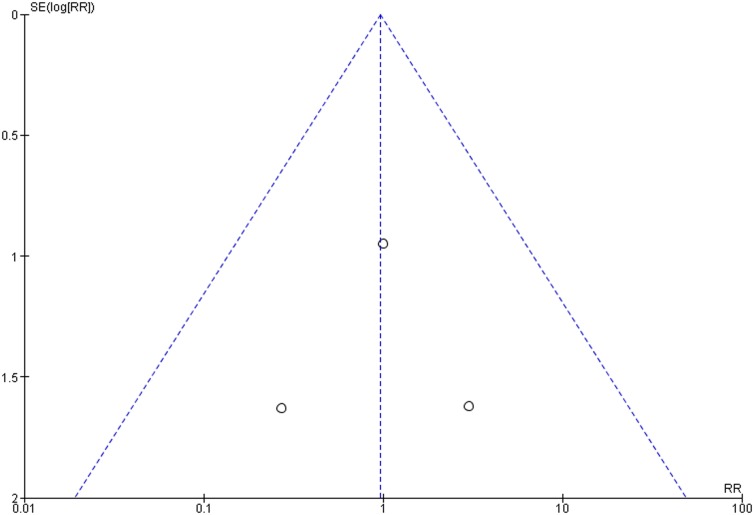
Funnel plot for the studies reporting the outcome event ‘postoperative infection’.

**Figure 7:**
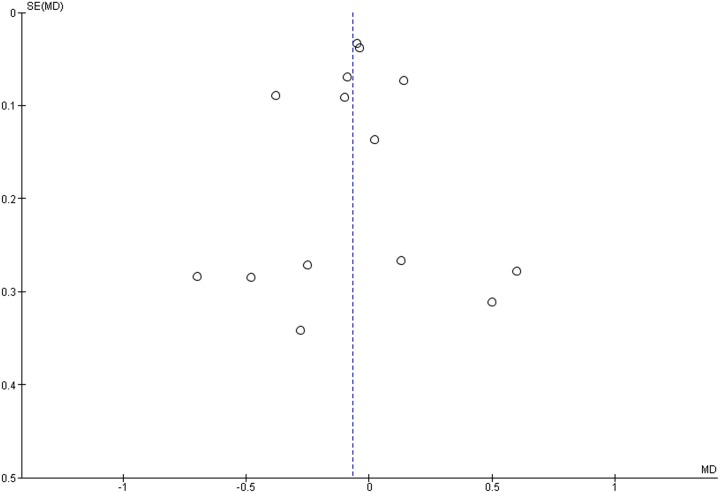
Funnel plot for the studies reporting the outcome event ‘marginal bone loss’.

The funnel plots did not show asymmetry when the studies reporting either the outcome ‘implant failure’ (Figure 5), ‘postoperative infection’ (Figure 6), or ‘marginal bone loss’ (Figure 7) are analyzed, indicating absence of publication bias.

## Discussion

Potential biases are likely to be greater for non-randomized studies compared with RCTs, so results should always be interpreted with caution when they are included in reviews and meta-analyses [8]. However, narrowing the inclusion criteria increases homogeneity but also excludes the results of more trials and thus risks the exclusion of significant data [29]. This was the reason to include non-randomized studies in the present meta-analysis. The issue is important because meta-analyses are frequently conducted on a limited number of RCTs. In meta-analyses such as these, adding more information from observational studies may aid in clinical reasoning and establish a more solid foundation for causal inferences [29].

The relevant question is whether the lack of a difference between the flapless and the open flap implant procedures in some studies concerning implant failure rates is a real finding or is due to the lack of statistical power, given the small number of patients per group in many studies [1], [4], [5], [11], [13], [15], [16]–[20], [22], [24]. However, there was a statistically and clinically significant difference (*P* = 0.03) favoring the open flap surgery was found after the meta-analyses, stressing the importance of meta-analyses to increase sample size of individual trials to reach more precise estimates of the effects of interventions. However, when studies of low and high risk of bias were pooled separately, there was a difference in the results and in the statistical significance of the RR. Thus, the results must be interpreted carefully.

One drawback found in six studies [3], [11], [12], [16], [17], [21] is the fact that the patients were followed for a short period (1–6 months). Thus, only early failures could be assessed. A longer follow-up period can lead to an increase in the failure rate, especially if it extended beyond functional loading, because other prosthetic factors can influence implant failure from that point onward [30]. The success of a dental implant should be defined after a minimal period of 12 months of implant loading. Early, intermediate, and long-term success has been suggested to span 1 to 3 years, 3 to 7 years, and more than 7 years respectively [31]. Moreover, the results found in the studies differed from each other, and this difference could be due to factors such as differences in the patients included in the study or the clinicians placing and restoring the implants.

The immediate loading only in the flapless group in some studies [10], [13] is a confounding factor, as well as the presence of smokers among the patients in several trials [5], [10], [14], [21]–[24], [27], the use of grafts [11], [24], [25], the use or not use of surgical guides, different prosthetic configurations, and the insertion of implants from different brands and surface treatments. Titanium with different surface modifications shows a wide range of chemical, physical properties, and surface topographies or morphologies, depending on how they are prepared and handled [32]–[34], and it is not clear whether, in general, one surface modification is better than another [35]. Also, there is the fact that some studies inserted some [14], [23], [25] or all implants in fresh extraction sockets [19]. One of these studies [19] placed all implants in infected extraction sockets. Moreover, in four studies [5], [19], [20], [28] flapless surgery was only considered in favorable clinical conditions. Flapless surgery was considered a treatment option based on clinical examination and largely depending on the anatomical condition of the bone after clinical and radiographic inspection. Therefore, the allocation to the surgical approach was biased (selection bias), what could have masked substantial intergroup disparity.

Currently, some software systems using computed tomography scans have been proposed to aid in planning surgery and to produce surgical drilling guides to transfer the planned position to the surgical field. These guides are manufactured in such a way that they match the location, trajectory, and depth of the planned implant with a high degree of precision. As the dental practitioner places the implants, the guides stabilize the drilling by restricting the degrees of freedom of the drill trajectory and depth [36]. It was stated that by using computer-assisted surgery predictability, precision and safety in flapless dental implantology are ensured [12].

However, the precision of the whole procedure depends largely on the ability to position accurately the drill guide, and to maintain that stable position during the whole procedure [36]. In the case of the placement of implants in completely edentulous jaws, there must be a way to assure the stability of the drill guide, and this is done by fixing the surgical guide onto the bone by osteosynthesis screws. Asymmetric distribution of the screws or uneven tightening of the screws could bring the drilling template out of balance. Furthermore, a certain error is induced as the diameter of the steel tubes is slightly larger than the drill diameter [36]. Finally, the largest error is probably due to the fact that the final step in the procedure is carried out manually, depending on the surgical guide used. In these cases, implant placement cannot be done through the surgical drill guide because of present mechanical limitations. The drill guide, therefore, has to be removed before the implant is actually inserted, leaving the possibility of additional deviation [36]. Because of these reasons, the surgical drill guide may provide a false security in decreasing the risk of bone fenestrations or perforations. This may be one of the reasons why it was observed, in the present review, a higher percentage of implant failures with the flapless technique when compared with the open flap surgery.

Still concerning the precision of the implant insertion, it is worth commenting about the technique used by the study of Sennerby et al. [25]. They made use of a slide-over guide sleeve to evaluate and determine the position of the implant. This system is based on the surgeons' imprecise opinion of what is the exact direction of the implants to be placed and it is subjected to flaws, which may have led to an increasing incidence of implant bone plate fenestrations or perforations, and consequently higher implant failure in this group (7.9% versus no failure in the open flap surgery). Correct bur angulation is critical in the procedure [36]. With the CT-guide surgery, it is possible to verify in advance the presence of concavities of the vestibular and lingual/palatal bone plates surrounding the planned implant surgical site, thus planning the correct bur angulation and decreasing the chance of implant bone fenestrations or perforations.

Moreover, one *in vitro* study [37] analyzed deviations in position and inclination of implants placed with flapless surgery compared with the ideally planned position and examined whether the outcome was affected by the experience level. The authors observed that the three-dimensional location of implants installed with flapless approach differed significantly from the ideal, although neighboring teeth were present and maximal radiographical information was available, and the outcome was not influenced by the level of experience with implant surgery. It was suggested that these deviations would in a clinical situation lead to complications such as loss of implant stability, aesthetical and phonetical consequences. The authors recommended the performance of more precise measurements of soft tissue *in situ* or additional use of guiding systems.

Since flapless implant placement generally is a “blind” surgical technique, care must be taken when placing implants. Angulation of the implants affected by drilling is critical so as to avoid perforation of the cortical plates, both lingual and buccal, especially on the lingual in the mandibular molar area and the anterior maxilla [38]. Therefore, the surgeon must weigh the benefits of the flapless technique in front of the increasing risk of implant bone fenestrations or perforations, which allegedly may impair implant success or increase the implant failure rates [39]. Violation of the dental implant beyond the alveolar housing may result in infection and ultimate loss of the implant [40]. There should be no problem if the patient has been appropriately selected and an appropriate width of bone is available for implant placement [38]. Some authors [38] suggested a minimum of 7 mm of bone width and substantial training to use the appropriate technique.

Another hypothetical drawback of the flapless procedure is that it could interfere with osseointegration because of implant surface contamination and the deposition of epithelial and connective cells from the oral mucosa in the bone during surgical preparation [27].

On the other hand, a flapless procedure could have a positive effect on the early bone remodeling process, because during the surgical procedure, the bone remains covered by the periosteum. However, the strongly tightened surgical template used in to insert implants in totally edentulous jaws may hinder access of saline water and proper cooling during the drilling procedure, which could negatively influence the implant surrounding the bone and the remodeling process during healing [21].

Concerning the marginal bone loss, one may expect that the open flap surgery may cause higher marginal bone loss due to decreased supraperiosteal blood supply because of the raising the tissue flap during the surgical procedure. Studies have demonstrated that flap reflection often results in bone resorption around natural teeth [41]. However, it was showed in five studies that the flapless technique generated more marginal bone loss around the implants [5], [11], [13], [20], [28]. The authors of some of the articles here reviewed provided some reasonable explanations for this. De Bruyn et al. [5] suggested that this was probably caused in their study due to overdoing of the countersinking procedure. More extensive widening of the crestal bone was necessary to remove enough bone as to allow proper placement of the healing abutment. By countersinking wider and deeper, the coronal portion of the implant is not always in intimate contact with the bone. In the flapped sites, the countersinking procedure was more controlled according to the guidelines of the manufacturer because visual inspection *in situ* was possible. Rousseau [28] discussed that this is due to implants being installed blindly, and thus implants are installed more deeply with the flapless technique than with the open flap technique. Therefore, a portion of the transmucosal (supracrestal) part of the implant is slightly below the crestal bone level. Because the coronal part of the implant is smooth titanium, rearrangement of bone around the neck of the implant is normal. When an open flap technique is used, the implant is installed under visual control directly at the right crestal bone position. This results in less bone rearrangement around the implant neck [28]. The results found in the study of Van de Velde et al. [13] may be related to the fact that the implants inserted through the flapless technique were immediately loaded, whereas the implants inserted through open flap surgery were loaded only after 6 weeks.

The results of the present study have to be interpreted with caution because of its limitations. First of all, all confounding factors may have affected the long-term outcomes and not just the use of flapless or open flap surgery, and the impact of these variables on the implant survival rate, postoperative infection and marginal bone loss is difficult to estimate if these factors are not identified separately between the two different procedures in order to perform a meta-regression analysis. The lack of control of the confounding factors limited the potential to draw robust conclusions. Second, some of the included studies had a retrospective design, and the nature of a retrospective study inherently results in flaws. These problems were manifested by the gaps in information and incomplete records. Furthermore, all data rely on the accuracy of the original examination and documentation. Items may have been excluded in the initial examination or not recorded in the medical chart [42], [43].

The authors of the present study believe that, for a more definite conclusion, future double-blinded RCTs with larger patient samples are required to determine the real effect of flapless implant surgery on patient outcome variables.

## Conclusion

The difference between the procedures (flapless vs. flapped) statistically affected the implant failure rates. However, the results must be interpreted carefully, as a sensitivity analysis revealed differences when the groups of studies of high and low risk of bias were pooled separately. No statistically significant effects of open flap surgery or flapless surgery on the occurrence of postoperative infection and on the marginal bone loss were observed.

## Supporting Information

Checklist S1 **PRISMA checklist.**
(PDF)
